# SorGSD: updating and expanding the sorghum genome science database with new contents and tools

**DOI:** 10.1186/s13068-021-02016-7

**Published:** 2021-08-03

**Authors:** Yuanming Liu, Zhonghuang Wang, Xiaoyuan Wu, Junwei Zhu, Hong Luo, Dongmei Tian, Cuiping Li, Jingchu Luo, Wenming Zhao, Huaiqing Hao, Hai-Chun Jing

**Affiliations:** 1 Key Laboratory of Plant Resources, Institute of Botany, Chinese Academy of Sciences, Beijing, 100093 China; 2 Engineering Laboratory for Grass-Based Livestock Husbandry, Institute of Botany, Chinese Academy of Sciences, Beijing, 100093 China; 3 University of Chinese Academy of Sciences, Beijing, 100049 China; 4 China National Center for Bioinformation, Beijing, 100101 China; 5 National Genomics Data Center, Beijing Institute of Genomics, Chinese Academy of Sciences, Beijing, 100101 China; 6 CAS Key Laboratory of Genome Sciences and Information, Beijing Institute of Genomics, Chinese Academy of Sciences, Beijing, 100101 China; 7 College of Life Sciences and Center for Bioinformatics, Peking University, Beijing, 100871 China

**Keywords:** Sorghum, Bio-energy plant, Variation, SNPs, Small INDELs, Phenotype, Database

## Abstract

**Background:**

As the fifth major cereal crop originated from Africa, sorghum (*Sorghum bicolor*) has become a key C_4_ model organism for energy plant research. With the development of high-throughput detection technologies for various omics data, much multi-dimensional and multi-omics information has been accumulated for sorghum. Integrating this information may accelerate genetic research and improve molecular breeding for sorghum agronomic traits.

**Results:**

We updated the Sorghum Genome SNP Database (SorGSD) by adding new data, new features and renamed it to Sorghum Genome Science Database (SorGSD). In comparison with the original version SorGSD, which contains SNPs from 48 sorghum accessions mapped to the reference genome BTx623 (v2.1), the new version was expanded to 289 sorghum lines with both single nucleotide polymorphisms (SNPs) and small insertions/deletions (INDELs), which were aligned to the newly assembled and annotated sorghum genome BTx623 (v3.1). Moreover, phenotypic data and panicle pictures of critical accessions were provided in the new version. We implemented new tools including ID Conversion, Homologue Search and Genome Browser for analysis and updated the general information related to sorghum research, such as online sorghum resources and literature references. In addition, we deployed a new database infrastructure and redesigned a new user interface as one of the Genome Variation Map databases. The new version SorGSD is freely accessible online at http://ngdc.cncb.ac.cn/sorgsd/.

**Conclusions:**

SorGSD is a comprehensive integration with large-scale genomic variation, phenotypic information and incorporates online data analysis tools for data mining, genome navigation and analysis. We hope that SorGSD could provide a valuable resource for sorghum researchers to find variations they are interested in and generate customized high-throughput datasets for further analysis.

**Supplementary Information:**

The online version contains supplementary material available at 10.1186/s13068-021-02016-7.

## Background

Sorghum ranks fifth in cereal production and acreage behind maize, rice, wheat and barley (http://www.fao.org). It is cultivated in vast geographic areas in the Americas, Africa, Asia, and Oceania. Sorghum’s excellent agronomic and biological properties, such as heat and drought tolerance, make it a vital grain crop in marginal land for production without competing against other major food crops [1]. With the increase of world population and the decrease of water resources, sorghum will become the preferred food crop all over the world in the future. Furthermore, sorghum is not only harvested for grain, but also often used to produce syrup, grazing and biomass production [2].

As a model organism that carries out C_4_ photosynthesis, sorghum was the second sequenced cereal crop after the C_3_ organism rice [3, 4]. The comparatively small genome of sorghum makes it a potential genetic model for the design of bioenergy crops compared with the larger and more repetitive genomes of other major C_4_ crops, such as maize and sugarcane. With the improvement of the reference genome (BTx623) [4, 5] and the development of sequencing technologies, studies on domestication and genetic mechanism of distinct phenotype in sorghum have been greatly accelerated [2, 6–17].

During the past decade, diverse web resources have been constructed to exhibit numerous omics data, which is beneficial for the sorghum research community (Table 1). Plant specific genome databases such as Phytozome [18] and Gramene [19], as well as the most comprehensive Genome OnLine Database (GOLD) [20] are widely used as data sources and analysis platforms for sorghum research. On the other hand, sorghum included plant secondary databases such as PIGD [21], PlanTFDB [22], DNApod [23], PceRBase [24], PtRFdb [25] and GreenPhylDB [26] have vital modules about sorghum resources. Finally, the sorghum specific secondary databases, including MOROKOSHI [27], PGSB [28], SorghumFDB [29], Sorghum QTL Atlas [30], and Sorghum Genomics, are a cluster of websites dedicated to sorghum researches. Among them, SorghumFDB is the most comprehensive sorghum specific database, which contains extensive public genomic and functional annotations data, as well as useful analysis tools. With published sorghum genome re-sequencing data of 48 accessions, we developed a sorghum SNP database (SorGSD) in 2016, providing the sorghum user community with abundant SNPs and some other resources related to sorghum genetics and genomics [31].

**Table 1:** Online databases for sorghum genome

Name	URL/description	PubMed ID
Comprehensive genome databases and analysis platforms		
Phytozome	https://phytozome.jgi.doe.gov/	[18]
	Plant genome database portal and analysis platform	22110026
Gramene	http://www.gramene.org/	[19]
	Plant genome database portal and analysis platform	33170273
GOLD	https://gold.jgi.doe.gov/	[20]
	Genomes online database	33152092
Sorghum included plant secondary databases		
PIGD	http://pigd.ahau.edu.cn/	[21]
	A database for intronless genes in *Poaceae*	25270086
PlantTFDB	http://planttfdb.gao-lab.org/	[22]
	A database of plant transcription factors	27924042
DNApod	http://tga.nig.ac.jp/dnapode	[23]
	DNA polymorphism annotation database	28234924
PceRBase	http://bis.zju.edu.cn/pcernadb/	[24]
	A database of plant competing endogenous RNA	28053167
PtRFdb	http://www.nipgr.res.in/PtRFdb	[25]
	A database for plant tRNA-derived fragments	29939244
GreenPhylDB	https://www.greenphyl.org/	[26]
	A comparative pangenomic database for plant genomes	33237299
Sorghum specific secondary databases		
MOROKOSHI	http://sorghum.riken.jp/	[27]
	Sorghum transcriptome database	25505007
SorGSD	http://sorgsd.big.ac.cn/	[31]
	Sorghum SNP database	26884811
PGSB	http://pgsb.helmholtz-muenchen.de/plant/sorghum/	[28]
	Plant genome and systems biology	26527721
SorghumFDB	http://structuralbiology.cau.edu.cn/sorghum/	[29]
	A database for sorghum functional genomics	27352859
Sorghum QTL Atlas	https://aussorgm.org.au/sorghum-qtl-atlas/	[30]
	Tool for searching QTL landscape in sorghum	30343386
Sorghum genomics	https://www.purdue.edu/sorghumgenomics/	N/A
	Functional Gene Discovery Platform for Sorghum	

Here, we announce and describe the second major release of the sorghum genome science database (SorGSD). The goal of the redesign is to construct a comprehensive database with sorghum genomic variations and phenotypes. Compared with the first version SorGSD which contains SNPs of 48 sorghum accessions, the second version provides a more extensive set of genomic variation data for both SNPs and small INDELs of 289 sorghum accessions, as well as characteristic phenotypic information and panicle pictures of critical sorghum lines. We also provide three useful tools in the new release, including ID Conversion, Homologue Search and Genome Browser. The back-end database framework and the web interface were redesigned as a part of the Genome Variation Map at the National Genomics Data Center (NGDC) and China National Center for Bioinformation (CNCB). We hope that these data and tools are beneficial for exploring genetic variations and evolution studies of sorghum and other species. The new version SorGSD is freely accessible at http://ngdc.cncb.ac.cn/sorgsd/.

## Results and discussion

### New data contents

The new version SorGSD was mainly built on sorghum reference genome BTx623 (v3.1) with improved assembly and gene annotations [5]. Currently, SorGSD contains 33,825,236 SNPs and 5,722,385 small INDELs identified from the re-sequencing data of 289 sorghum lines [6, 32, 33], including three accessions of *Sorghum propinquum*, 50 wild/weedy sorghums and 236 cultivated sorghums (Additional file 1: Table S1). After annotation and calculation, we obtained detailed information about the distribution of variations in different genomic regions, including genic, intergenic, and intronic regions (Table 2). On the other hand, we also collected about 70 kinds of phenotypic data over 183 accessions with plant ID (PI) from the U.S. National Plant Germplasm System (GRIN-Global) and panicle pictures of 174 critical accessions taken in our laboratory. Besides, we renewed the introduction about sorghum genome, sorghum resources websites including general information, genome and transcriptome databases, research institutions and sorghum producers around the world, as well as critical references about sorghum genetics and genomics.

**Table 2:** Distribution of variations in different genomic regions

Consequence type	SNPs	Small INDELs
Intergenic	20,683,922	2,248,312
Upstream	12,974,750	4,145,728
Downstream	11,903,259	3,904,589
Intron	4,263,151	1,386,417
5′ UTR	4,89,036	2,72,597
3′ UTR	8,05,777	3,13,469
Missense	7,84,695	–
Synonymous	6,67,205	–
Splice region	1,00,793	33,979
Start lost	2152	849
Stop lost	1813	722
Stop gained	16,102	4730
Stop retained	1002	409
Splice acceptor	4160	3292
Splice donor	3686	3858
Coding sequence	89	4480
Inframe deletion	–	52,218
Inframe insertion	–	41,617
Frameshift	–	1,04,763
Protein altering	–	3656

### New features of the database

**Fig. 1:**
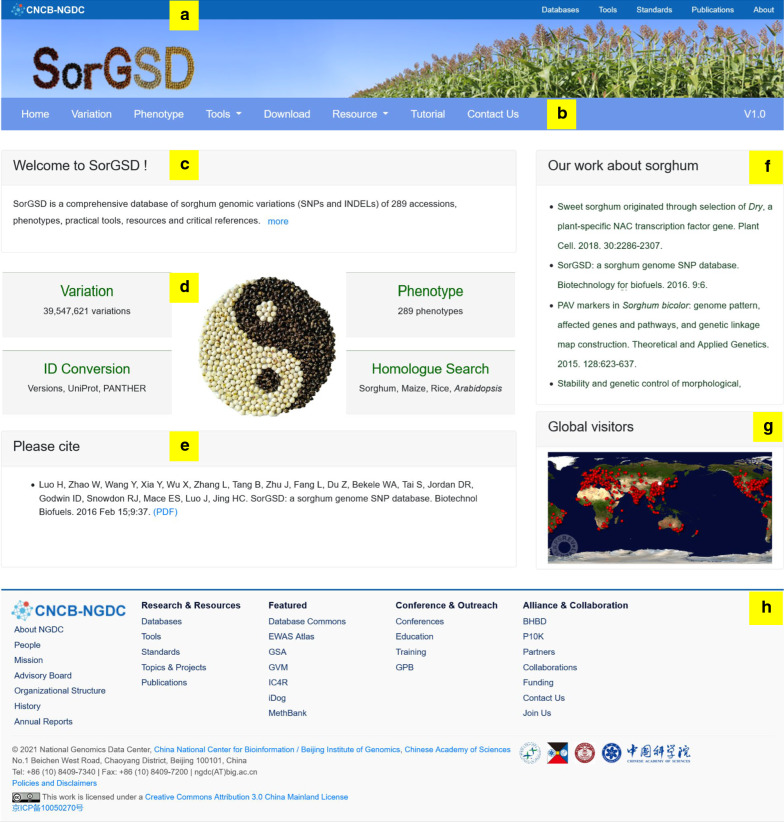
Schematic diagram of the SorGSD home page. The background of CNCB-NGDC is shown in **a** and **h**. The menu bar (**b**), welcome message (**c**), shortcuts of core functions (**d**) and prompt of citation (**e**) are placed from up to bottom. Our laboratory’s major publications (**f**) and website browsing history (**g**) could be acquired on the right side

SorGSD is free and open to the public with comprehensive functions (Fig. 1; Additional file 2: Table S2). In this update, we put the main page under the National Genomics Data Center of the China National Center for Bioinformation (CNCB-NGDC) (Fig. 1a, h) [34]. Links to each page are shown at the menu bar (Fig. 1b), and a simple welcome message is displayed under the menu bar (Fig. 1c). Four shortcuts of core functions and prompt of citation can be found on the home page (Fig. 1d, e). Our laboratory’s major publications and website browsing history could be acquired easily on the right side (Fig. 1f, g).

It is worth mentioning that we still keep the original version up and running, and users could browse it by clicking the “V1.0” button on the menu bar and switch back to the new version by clicking the “V2.0” button of the old version. We optimized the presentation interface to make it easier for users to search for variations. Phenotypic details of each accession could be searched directly. The browsing interface of critical references was redesigned for a better user experience. We also provided three new tools: ID conversion, Homologue Search and Genome Browser. Online documentation is provided to help users get familiar with the database. More detailed information is described as follows.

#### Improved variation search function

**Fig. 2:**
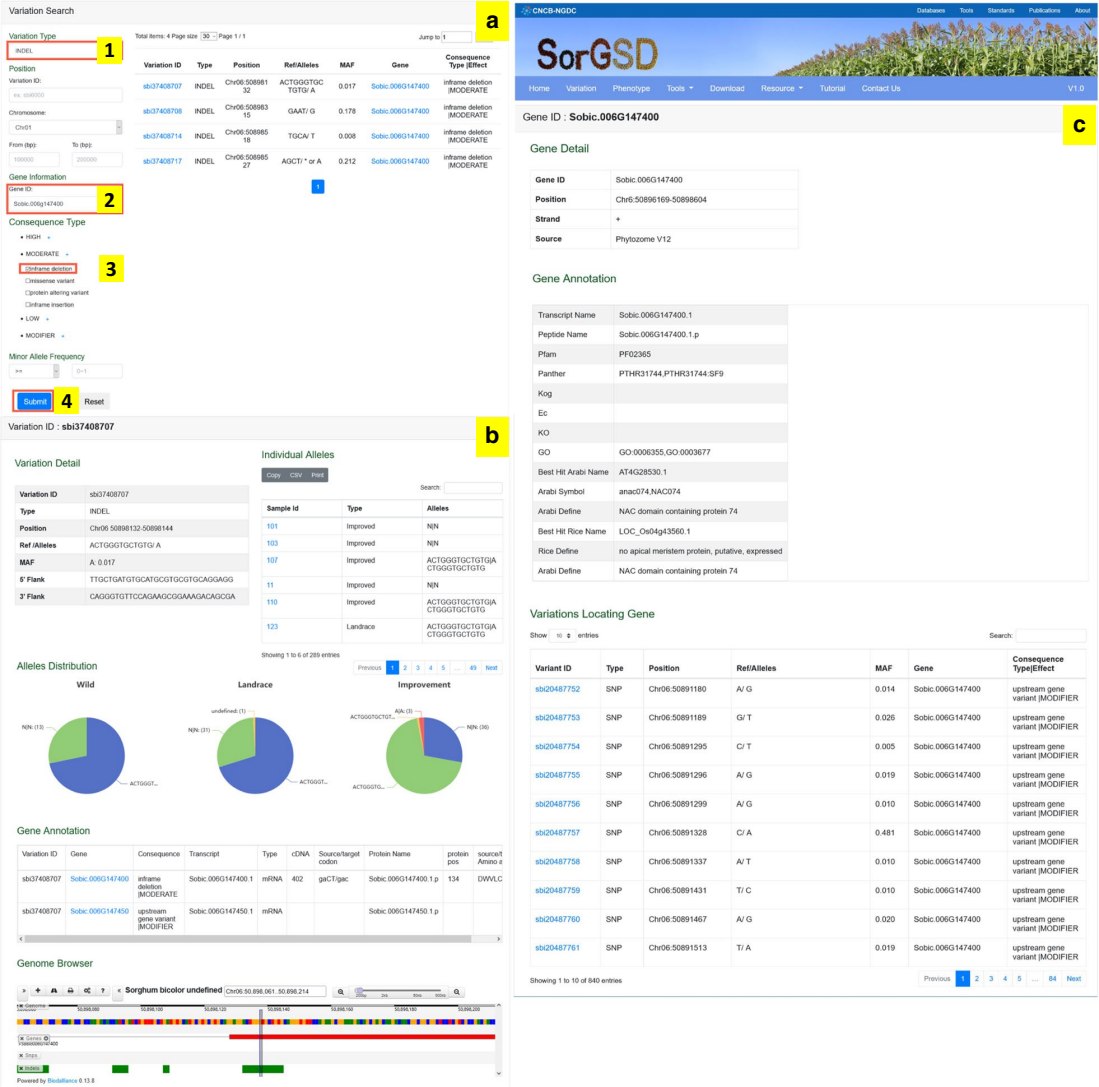
Steps and results of variation search. **a**. The search page of variations. Numbers in **a** show the steps of the search. **b.** Detail page of the target variation. **c.** Detail page of the gene with target variations

Users may search variation by typing in the variation type, genome position or gene ID. Furthermore, it is also possible to filter variation through consequence type and minor allele frequency (MAF) value. In our previous work, we found that the *Dry* gene encoded a plant-specific NAC transcription factor, which had a few loss-of-function mutations in sweet sorghum [33]. An inframe deletion variation (Chr06:50898132) within the conserved functional NAC domain could turn pithy stem into juicy stem, which is one reason for the origin of sweet sorghum. Here we take the *Dry* gene as an example to search this inframe deletion (Chr06:50898132). Firstly, we can enter the “Variation Search” page and choose the variation type as “INDELs”; secondly, type the gene ID of version 3.1 (*Sobic.006g147400*) in the edit box “Gene ID”; thirdly, tick “inframe deletion” in “MODERATE” under “Consequence Type”; finally, click “Submit” and we can get the list of target small INDELs at the region of *Dry* on the right hand of the page (Fig. 2a).

In the list, we could see that the first one is the target small INDELs we searched (Fig. 2a). The details of the variation could be obtained by clicking the variation ID. Users may browse the no-redundant and individual variations with text format in three tables, one alleles distribution diagram and the chromosome-based graphical Genome Browser interface (Fig. 2b). In the text format tables, variation details (e.g., chromosome location, reference allele and three-fifths flank sequences), individual alleles and details of the annotated gene of the variation are given. The alleles distribution diagram is used to infer evolutionary scenario of each variation during sorghum domestication and improvement. More importantly, the individual alleles of target variation can be downloaded to perform subsequent analysis, such as phylogenetic tree construction and association analysis. Users can enter the gene page by clicking the gene ID with a blue background in the “Gene Annotation” table. The gene detail, gene annotation and all the variations locating gene, including SNPs and small INDELs without filtered, will be listed in three tables, respectively (Fig. 2c).

On the other hand, the demand of searching all the SNPs in the position of *Dry* could be obtained on the “Variation Search” page (Fig. 2a) by the following steps: (1) choose the variation type as “SNP”; (2) choose the chromosome as “Chr06”; (3) input the physical location (Chr06:50896169.50898604) and submit, we can get all the SNPs in the site of *Dry*.

#### New phenotype search function

**Fig. 3:**
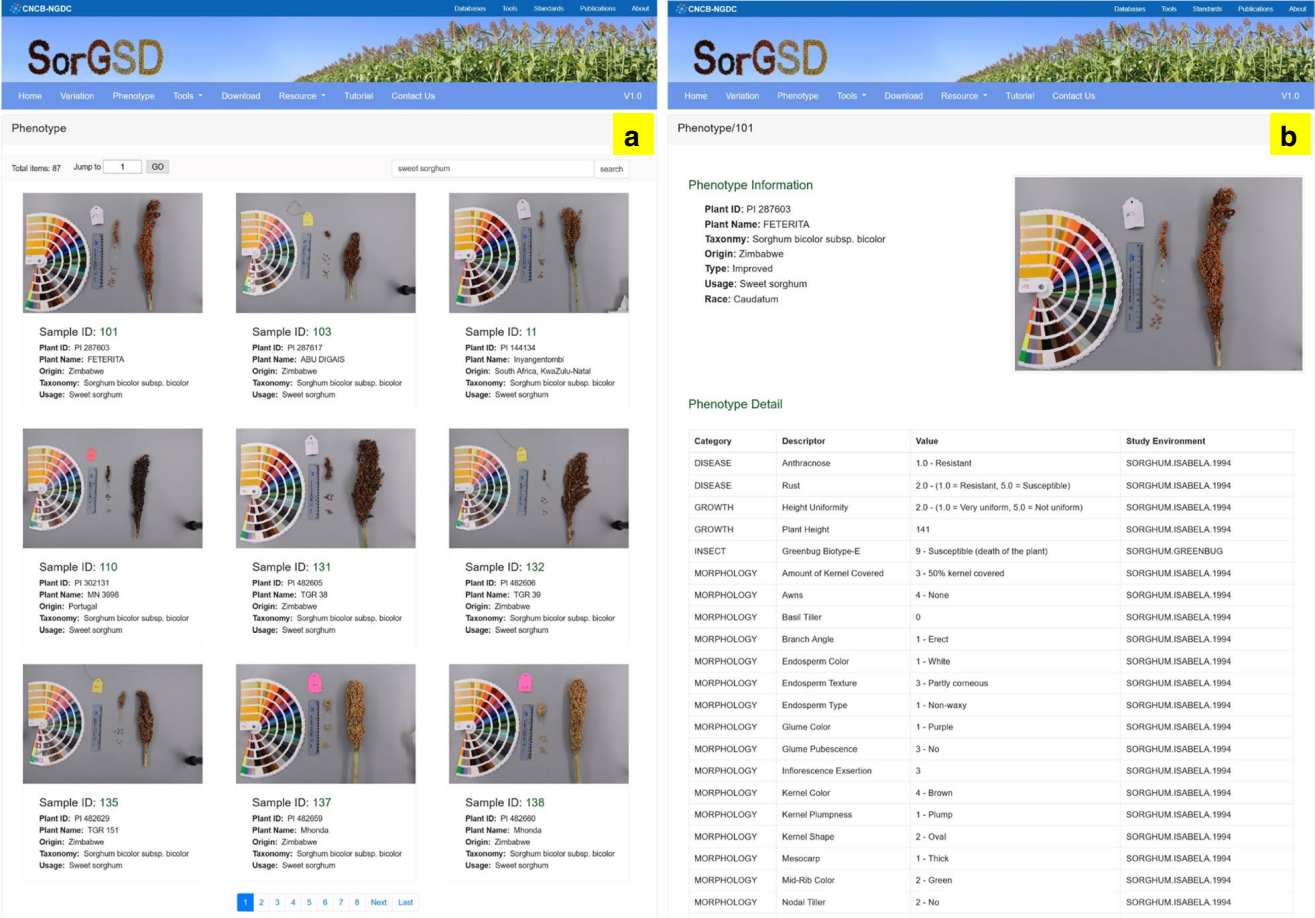
Searching page (**a**) of accessions and result page (**b**) of the target accession

A user-friendly web interface is provided for users to browse and retrieve phenotypic information (Fig. 3). On this page, users can search for important information of samples using several keywords, including sample ID, plant ID, plant name, origin, taxonomy and usage. When we input “sweet sorghum” in the search box, we can obtain all accessions with the keyword of individual information (Fig. 3a). A high-resolution image could be exhibited by clicking each sample’s picture to see the detail of panicle and seed appearance. For example, sample “101” is an improved sweet sorghum from Zimbabwe. By clicking the “Sample ID: 101” tab, the result page will list all agronomic traits’ values (Fig. 3b). It is noteworthy that users could also enter the phenotypic page to view the value of this trait from the variation detail page by clicking the tab of “Sample ID” in the “Individual Alleles” table (Fig. 2b).

#### New online tool

SorGSD provides three online tools (e.g., ID Conversion, Homologue Search and Genome Browser) for users to analyze their data. ID Conversion is a useful tool to convert sorghum gene IDs from one to other ID systems of v1.4, v2.1 and v3.1, as well as the IDs of UniProt and PANTHER databases. When we type the gene ID (v3.1) of *Dry* gene (*Sobic.006g147400*) in the search box and press “Convert”, the corresponding ID of other versions and systems will be listed in the result table. Users could access directly to the corresponding pages of the IDs of UniProt and PANTHER through the hyperlink.

To better understand the evolution of sorghum genes, Homologue Search is built to identify homologous genes among sorghum, maize, rice and *Arabidopsis*. When we input the gene ID of *Dry* gene (*Sobic.006g147400*) in the “Gene Name” box and click “Submit”, the list of homologues in other species will be displayed. Besides, we provided a Genome Browser to visualize the locus of variation in the genome. Users only need to type in the genome position (e.g., *Dry* gene, Chr06:50896169.50898604), corresponding transcript information of the gene and the positions of SNPs and INDELs in the relevant range will appear on the results page. We also provided the link to BLAST tool rested on CNCB-NGDC for comparing nucleotide or protein sequences with sorghum reference sequence database.

#### Revised resource page

The resource page is divided into three sections, including “Genome”, “Website” and “Reference”. The “Genome” part introduces the general information of sorghum genome. Users could enter the homepages of website resources promptly on the “Website” page. It is worth mentioning that we updated 162 vital publications of sorghum and classed them into six broad categories in “Reference”. By clicking the class title heading in the directory on the left of the page, all papers in the target category will be listed on the right hand. Consumers could read the abstract or download the article from the links by clicking the button “Abstract”.

## Conclusions and future directions

SorGSD is committed to providing a wide range of sorghum genome data, including genomic information, detailed phenotypic data, sorghum resources and analysis tools for sorghum scientists and breeders. The interface of new version SorGSD is under the CNCB-NGDC and also an essential part of the Genome Variation Map (GVM), a data repository of genome variations of human, as well as cultivated plants and domesticated animals [35]. In this upgrade, we added 241 varieties of whole-genome variation data (including SNPs and small INDELs) based on the latest sorghum reference annotation (version 3.1). The total number of accessions (289) and variations (39.5 Mb) are 6 times and 1.4 times as much as that of the first version, respectively. We also added about 70 kinds of traits information of 183 accessions, which provides detailed reference data of each line for breeders. Tools of ID Conversion, Homologue Search and Genome Browser provide visual, convenient and quick queries for scientific workers engaged in sorghum study. Besides, we carried out a brand new page design to optimize the user experience and make the interaction friendlier. The simple and straight forward user guide allows users to be familiar with the web page’s overall design and realize various functions of the webpage quickly.

In the future, we will update SorGSD regularly and add variations with newly available re-sequenced sorghum accessions. In the next step, we anticipate integrating phenotypic data, genomic variation data, transcriptome data, proteome data, and epigenomic data, as well as metabolomics and metabolic interaction networks to build a comprehensive sorghum research and analysis database. At the same time, we hope to receive comments and suggestions, aiming to make SorGSD a one-stop sorghum research platform with multi-faceted omics data and analysis tool.

## Methods and materials

### Data resources

Currently, we collected the re-sequencing data with the unique average depth of 4.02–48.55 ×  coverage from three sets of sorghum germplasms comprising a total of 289 accessions of wild and cultivated sorghum. The most extensive set of germplasm is a diverse panel of 241 sorghum lines which we published to explore the origin of sweet sorghum through the selection of *Dry* gene [33]. The second dataset is 44 sorghum lines which revealed untapped genetic potential in Africa’s indigenous cereal crop sorghum by Jordan’s Lab in 2013 [6]. The last dataset is also our group’s work which contains three accessions of cultivated sorghums [32]. The entire set of original sequence data could be obtained from Genome Sequence Archive [36]. Phenotypic data cover the breed and agronomic-trait information collected from GRIN-Global (npgsweb.ars-grin.gov/). Finally, panicle pictures were taken when the sorghum plant reached maturity in the experimental fields of the Institute of Botany, Chinese Academy of Sciences (Beijing, China) in 2019.

### Data processing

After trimming the adapter and filtering low-quality reads of the second [6] and third [32] datasets in the first dataset [33], the remaining clean reads were mapped to the reference genome BTx623 (v3.1) with BWA (v0.7.8) [37]. The mapping results were converted to BAM format, and the duplicated reads and multi-aligned reads were eliminated by the SAMtools package (v1.3) [38]. GVCF files of these lines were generated by *HaplotypeCaller* in GATK (v3.1) [39]. All the GVCF files of the three datasets were used to call SNPs and INDELs by *GenotypeGVCFs* in GATK (v3.1) [39]. In total, 33,825,236 SNPs and 5,722,385 small INDELs were identified across 289 sorghum lines. Finally, we predicted and annotated the effects of variations by using the VEP program (v84) [40]. Besides, we also calculated the MAF of each variant using vcftools (v0.1.13) [41].

### Database design and implementation

SorGSD was designed based on the framework of the iDog database [42], which was implemented using Spring Boot (http://sping.io), a free and prevailing Model-View-Controller (MVC) framework, and Mybatis (https://mybatis.org/mybatis-3/), a first-class persistence framework with support for custom SQL, stored procedures and advanced mappings. In the back-end part, metadata and reference data were stored in MySQL (https://www.mysql.com). Web user interfaces were developed using JSP, JQuery as well as BootStrap. The Biodalliance genome browser (http://www.biodalliance.org/) was used for genome synteny visualization.

## Supplementary Information

**Additional file 1: Table S1.** Information of 289 sorghum accessions.

**Additional file 2: Table S2.** Feature comparisons between two versions.

## Data Availability

All datasets are available at http://ngdc.cncb.ac.cn/sorgsd/download.

## Abbreviations

SNP: Single nucleotide polymorphism
INDEL: Insertion/deletion
NGDC: National Genomics Data Center
CNCB: China National Center for Bioinformation
PI: Plant ID
MAF: Minor allele frequency
GVM: Genome variation map
BWA: Burrows–Wheeler alignment
GATK: Genome analysis toolkit
VEP: Variant effect predictor
MVC: Model-View-Controller
SQL: Structured query language
JSP: Java server pages

## References

[1] Hao HQ, Li ZG, Leng CY, Lu C, Luo H, Liu YM, Wu XY, Liu ZQ, Shang L, Jing HC (2021). Sorghum breeding in the genomic era: opportunities and challenges. Theor Appl Genet.

[2] Boyles RE, Brenton ZW, Kresovich S (2019). Genetic and genomic resources of sorghum to connect genotype with phenotype in contrasting environments. Plant J.

[3] Sorghum Genomics Planning Workshop p (2005). Toward sequencing the sorghum genome. A U.S. National Science Foundation-sponsored workshop report. Plant Physiol.

[4] Paterson AH, Bowers JE, Bruggmann R, Dubchak I, Grimwood J, Gundlach H, Haberer G, Hellsten U, Mitros T, Poliakov A (2009). The *Sorghum bicolor* genome and the diversification of grasses. Nature.

[5] McCormick RF, Truong SK, Sreedasyam A, Jenkins J, Shu S, Sims D, Kennedy M, Amirebrahimi M, Weers BD, McKinley B (2018). The *Sorghum bicolor* reference genome: improved assembly, gene annotations, a transcriptome atlas, and signatures of genome organization. Plant J.

[6] Mace ES, Tai SS, Gilding EK, Li YH, Prentis PJ, Bian L, Campbell BC, Hu WS, Innes DJ, Han XL (2013). Whole-genome sequencing reveals untapped genetic potential in Africa’s indigenous cereal crop sorghum. Nat Commun.

[7] Morris GP, Rhodes DH, Brenton Z, Ramu P, Thayil VM, Deshpande S, Hash CT, Acharya C, Mitchell SE, Buckler ES (2013). Dissecting genome-wide association signals for loss-of-function phenotypes in sorghum flavonoid pigmentation traits. G3.

[8] Morris GP, Ramu P, Deshpande SP, Hash CT, Shah T, Upadhyaya HD, Riera-Lizarazu O, Brown PJ, Acharya CB, Mitchell SE (2013). Population genomic and genome-wide association studies of agroclimatic traits in sorghum. Proc Natl Acad Sci USA.

[9] Thurber CS, Ma JM, Higgins RH, Brown PJ (2013). Retrospective genomic analysis of sorghum adaptation to temperate-zone grain production. Genome Biol.

[10] Hayes CM, Burow GB, Brown PJ, Thurber C, Xin ZG, Burke JJ (2015). Natural variation in synthesis and catabolism genes influences dhurrin content in sorghum. Plant Genome.

[11] Anami SE, Zhang LM, Xia Y, Zhang YM, Liu ZQ, Jing HC (2015). Sweet sorghum ideotypes: genetic improvement of the biofuel syndrome. Food Energy Secur.

[12] Anami SE, Zhang LM, Xia Y, Zhang YM, Liu ZQ, Jing HC (2015). Sweet sorghum ideotypes: genetic improvement of stress tolerance. Food Energy Secur.

[13] Boyles RE, Cooper EA, Myers MT, Brenton Z, Rauh BL, Morris GP, Kresovich S (2016). Genome-wide association studies of grain yield components in diverse sorghum germplasm. Plant Genome.

[14] Brenton ZW, Cooper EA, Myers MT, Boyles RE, Shakoor N, Zielinski KJ, Rauh BL, Bridges WC, Morris GP, Kresovich S (2016). A genomic resource for the development, improvement, and exploitation of sorghum for bioenergy. Genetics.

[15] Maina F, Bouchet S, Marla SR, Hu Z, Morris GP (2018). Population genomics of sorghum (*Sorghum bicolor*) across diverse agroclimatic zones of Niger. Genome.

[16] Tao YF, Zhao XR, Wang XM, Hathorn A, Hunt C, Cruickshank AW, van Oosterom EJ, Godwin ID, Mace ES, Jordan DR (2020). Large-scale GWAS in sorghum reveals common genetic control of grain size among cereals. Plant Biotechnol J.

[17] Tao YF, Luo H, Xu JB, Cruickshank A, Zhao XR, Teng F, Hathorn A, Wu XY, Liu YM, Shatte T (2021). Extensive variation within the pan-genome of cultivated and wild sorghum. Nat Plants.

[18] Goodstein DM, Shu S, Howson R, Neupane R, Hayes RD, Fazo J, Mitros T, Dirks W, Hellsten U, Putnam N, Rokhsar DS (2012). Phytozome: a comparative platform for green plant genomics. Nucleic Acids Res.

[19] Tello-Ruiz MK, Naithani S, Gupta P, Olson A, Wei S, Preece J, Jiao Y, Wang B, Chougule K, Garg P (2021). Gramene 2021: harnessing the power of comparative genomics and pathways for plant research. Nucleic Acids Res.

[20] Mukherjee S, Stamatis D, Bertsch J, Ovchinnikova G, Sundaramurthi JC, Lee J, Kandimalla M, Chen IA, Kyrpides NC, Reddy TBK (2021). Genomes OnLine Database (GOLD) vol 8: overview and updates. Nucleic Acids Res.

[21] Yan HW, Jiang CP, Li XY, Sheng L, Dong Q, Peng XJ, Li Q, Zhao Y, Jiang HY, Cheng BJ (2014). PIGD: a database for intronless genes in the *Poaceae*. BMC Genomics.

[22] Jin J, Tian F, Yang DC, Meng YQ, Kong L, Luo J, Gao G (2017). PlantTFDB 40: toward a central hub for transcription factors and regulatory interactions in plants. Nucleic Acids Res.

[23] Mochizuki T, Tanizawa Y, Fujisawa T, Ohta T, Nikoh N, Shimizu T, Toyoda A, Fujiyama A, Kurata N, Nagasaki H (2017). DNApod: DNA polymorphism annotation database from next-generation sequence read archives. PLoS ONE.

[24] Yuan CH, Meng XW, Li X, Illing N, Ingle RA, Wang JJ, Chen M (2017). PceRBase: a database of plant competing endogenous RNA. Nucleic Acids Res.

[25] Gupta N, Singh A, Zahra S, Kumar S (2018). PtRFdb: a database for plant transfer RNA-derived fragments. Database.

[26] Valentin G, Abdel T, Gaetan D, Jean-Francois D, Matthieu C, Mathieu R (2021). GreenPhylDB v5: a comparative pangenomic database for plant genomes. Nucleic Acids Res.

[27] Makita Y, Shimada S, Kawashima M, Kondou-Kuriyama T, Toyoda T, Matsui M (2015). MOROKOSHI: transcriptome database in *Sorghum bicolor*. Plant Cell Physiol.

[28] Spannagl M, Nussbaumer T, Bader KC, Martis MM, Seidel M, Kugler KG, Gundlach H, Mayer KF (2016). PGSB PlantsDB: updates to the database framework for comparative plant genome research. Nucleic Acids Res.

[29] Tian T, You Q, Zhang LW, Yi X, Yan HY, Xu WY, Su Z (2016). SorghumFDB: sorghum functional genomics database with multidimensional network analysis. Database.

[30] Mace E, Innes D, Hunt C, Wang XM, Tao YF, Baxter J, Hassall M, Hathorn A, Jordan D (2019). The sorghum QTL atlas: a powerful tool for trait dissection, comparative genomics and crop improvement. Theor Appl Genet.

[31] Luo H, Zhao WM, Wang YQ, Xia Y, Wu XY, Zhang LM, Tang BX, Zhu JW, Fang L, Du ZL (2016). Erratum to: SorGSD: a sorghum genome SNP database. Biotechnol Biofuels.

[32] Zheng LY, Guo XS, He B, Sun LJ, Peng Y, Dong SS, Liu TF, Jiang S, Ramachandran S, Liu CM, Jing HC (2011). Genome-wide patterns of genetic variation in sweet and grain sorghum (*Sorghum bicolor*). Genome Biol.

[33] Zhang LM, Leng CY, Luo H, Wu XY, Liu ZQ, Zhang YM, Zhang H, Xia Y, Shang L, Liu CM (2018). Sweet sorghum originated through selection of *Dry*, a plant-specific NAC transcription factor gene. Plant Cell.

[34] Members C-N, Partners (2021). Database resources of the National Genomics Data Center, China National Center for bioinformation in 2021. Nucleic Acids Res.

[35] Li CP, Tian DM, Tang BX, Liu XN, Teng XF, Zhao WM, Zhang Z, Song SH (2021). Genome Variation Map: a worldwide collection of genome variations across multiple species. Nucleic Acids Res.

[36] Wang YQ, Song FH, Zhu JW, Zhang SS, Yang YD, Chen TT, Tang BX, Dong LL, Ding N, Zhang Q (2017). GSA: genome sequence archive. Genom Proteom Bioinf.

[37] Li H, Durbin R (2009). Fast and accurate short read alignment with Burrows-Wheeler transform. Bioinformatics.

[38] Li H, Handsaker B, Wysoker A, Fennell T, Ruan J, Homer N, Marth G, Abecasis G, Durbin R (2009). Genome Project Data Processing S. The sequence alignment/map format and SAMtools. Bioinformatics.

[39] McKenna A, Hanna M, Banks E, Sivachenko A, Cibulskis K, Kernytsky A, Garimella K, Altshuler D, Gabriel S, Daly M, DePristo MA (2010). The Genome Analysis Toolkit: a mapreduce framework for analyzing next-generation DNA sequencing data. Genome Res.

[40] McLaren W, Gil L, Hunt SE, Riat HS, Ritchie GR, Thormann A, Flicek P, Cunningham F (2016). The ensembl variant effect predictor. Genome Biol.

[41] Danecek P, Auton A, Abecasis G, Albers CA, Banks E, DePristo MA, Handsaker RE, Lunter G, Marth GT, Sherry ST (2011). The variant call format and VCFtools. Bioinformatics.

[42] Tang BX, Zhou Q, Dong LL, Li WL, Zhang XQ, Lan L, Zhai S, Xiao JF, Zhang Z, Bao YM (2019). iDog: an integrated resource for domestic dogs and wild canids. Nucleic Acids Res.

