# Supplementary material for: SorGSD: updating and expanding the sorghum genome science database with new contents and tools
Source: Biotechnol Biofuels. 2021 Aug 3;14:165. doi: 10.1186/s13068-021-02016-7 (PMC8336335; doi:10.1186/s13068-021-02016-7)
Supplement: Supplementary file 2 — Additional file 2: Table S2. Feature comparisons between two versions. [file 13068_2021_2016_MOESM2_ESM.docx]

**Table S2.** Features comparison between two versions.

| **Features** | | **Version 1** | **Version 2** |
| --- | --- | --- | --- |
| Data contents | SNP variation | 28.8 million | 33.8 million |
|  | Small INDEL variation | not provided | 5.7 million |
|  | Phenotypic information | not provided | provided |
| Tools | ID Conversion | not provided | provided |
|  | Homologue Search | not provided | provided |
|  | Genome Browser | not provided | provided |
| Resources | Genome | provided | renovated |
|  | Website | provided | renovated |
|  | Reference | provided | renovated |
